# Delays in Human-Computer Interaction and Their Effects on Brain Activity

**DOI:** 10.1371/journal.pone.0146250

**Published:** 2016-01-08

**Authors:** Christin Kohrs, Nicole Angenstein, André Brechmann

**Affiliations:** Special lab Non-invasive Brain Imaging, Leibniz Institute for Neurobiology, Magdeburg, Germany; Sichuan University, CHINA

## Abstract

The temporal contingency of feedback is an essential requirement of successful human-computer interactions. The timing of feedback not only affects the behavior of a user but is also accompanied by changes in psychophysiology and neural activity. In three fMRI experiments we systematically studied the impact of delayed feedback on brain activity while subjects performed an auditory categorization task. In the first fMRI experiment, we analyzed the effects of rare and thus unexpected delays of different delay duration on brain activity. In the second experiment, we investigated if users can adapt to frequent delays. Therefore, delays were presented as often as immediate feedback. In a third experiment, the influence of interaction outage was analyzed by measuring the effect of infrequent omissions of feedback on brain activity. The results show that unexpected delays in feedback presentation compared to immediate feedback stronger activate inter alia bilateral the anterior insular cortex, the posterior medial frontal cortex, the left inferior parietal lobule and the right inferior frontal junction. The strength of this activation increases with the duration of the delay. Thus, delays interrupt the course of an interaction and trigger an orienting response that in turn activates brain regions of action control. If delays occur frequently, users can adapt, delays become expectable, and the brain activity in the observed network diminishes over the course of the interaction. However, introducing rare omissions of expected feedback reduces the system’s trustworthiness which leads to an increase in brain activity not only in response to such omissions but also following frequently occurring and thus expected delays.

## Introduction

The livelong experience of humans from interactions with other humans has led to expectations regarding the general rules of communication that are automatically applied to interactions with technical systems. One such fundamental rule is the expectation of the sender to obtain feedback that a message has been received, i.e. the subjective sense of completion of an action [1]. While in human conversation eye contact may already satisfy this expectation, technical systems are usually not equipped with comparable competences and must even more so ensure immediate responses to indicate that a users’ action has been processed. Even though the performance of (computer) systems has improved significantly with every decade, we still face unexpected delays. Not just because network-based computing gains importance in a growing number of mobile or internet applications, delays or even interruptions caused by network-related problems may disturb the interactions. These interruptions have a tremendous effect on the behavior of a user (see [2] for a review). Early on, specific response time guidelines recommend various system response times depending on the complexity of the interaction [1–3]. Especially in very simple, so called control tasks, systems should behave like physical devices and respond within less than a few tenth of a second [2]. Otherwise users get frustrated, annoyed or even angry [2,4]. The physiological reactions on delays in system response have been measured in several studies, showing for instance an increase in skin conductance [5,6] or changes in EEG components, like the P300 [7]. Studies from the 80s and 90s often used delays longer than one second, not that common in today’s systems. However, a recent study could show that even delays of only 500 ms activate a network of brain regions which reflects an increase in attentional demand and adjustments in cognitive and action control [8]. However, from those studies it is not clear how long such delays in simple but ubiquitous control tasks can actually be without triggering additional neural resources. Therefore, we systematically investigated the effect of delayed feedback on brain activity of subjects engaged in a simple categorization task. In a first experiment we measured the differential impact of three delay durations (200 ms, 400 ms and 600 ms), followed by measuring the just noticeable delay of our participants under the given experimental conditions. In a second experiment we changed the probability of occurrence of such delays and investigated if users can adapt to frequently occurring delays and if this process is reflected by an adaptation in brain activity. Finally, we questioned which circumstances might negatively affect this adaptation. Systems that fail to respond–due to network problems or internal errors—might lose the users’ confidence into the system. Therefore, we hypothesize that occasional interruptions in user-system communications have an adverse influence on the effect of mere delays in system response.

## Materials and Methods

### Participants

In the first fMRI experiment, 31 right-handed subjects [Edinburgh Handedness Inventory, [9]] participated. Eight participants were excluded from further analysis because of excessive head motion (more than 3°or 2.5 mm) according to the recommendation given in [10]. This large proportion of drop-outs is possibly related to the duration of the fMRI scan of 45 minutes which for some subjects was too long to lie still. Another two participants were excluded due to eddy current quality after a maintenance of the scanner by the manufacturer. Thus, the data of 21 participants (10 females and 11 males, aged 19–30 years, mean age 24.5 ±2.5 years) were used for the group level analysis. Subsequent to the fMRI session (at the latest after two weeks), the individual just noticeable delay of each participant was measured.

In the second fMRI experiment 20 right handed subjects participated. One participant was excluded from further analysis because of motion artifacts. Thus, the data of 19 participants (7 females and 12 males, aged 21–32 years, mean age 26.3 ±3.5 years) were used for the group level analysis.

In the third fMRI experiment 22 right handed subjects participated. Five participants were excluded from further analysis because of motion artifacts. Thus, the data of 17 participants (9 females and 8 males, aged 22–38 years, mean age 27.2 ±3.8 years) were used for the group level analysis.

All participants gave written informed consent to the study that was approved by the ethics committee of the University of Magdeburg. The participants of the fMRI experiments were naïve with regard to the occurrence of delayed feedback trials. Before the third fMRI experiment subjects were told that technical problems may cause an omission of feedback in some trials to prevent them from stopping the whole experiment, however, nothing was told about the delay trials. Subsequent to the fMRI experiments, participants had to answer a questionnaire, which examined if participants detected the delays during the measurement. Only at this time participants were informed about the occurrence of delays and the aim of the experiment. None of the subjects was measured twice in the fMRI experiments.

### Stimuli and task

In all experiments participants had to solve an auditory categorization task. Upward and downward frequency modulated (FM) tones served as acoustic stimuli. These FM tones differed in center frequency (F_C_ = 700 Hz to 3800 Hz in steps of 100Hz), the starting and end frequency was calculated by F_C_[Hz] ± F_C_[Hz]/k * tone duration[s]. In the first experiment the FM tones were modulated by k = 2, 4, or 6 and hat a duration of 400 ms. In the second and third fMRI experiment the duration of the tones was 600 ms and they were modulated with k = 2. During the fMRI experiments the stimuli were presented pseudo-randomly with an inter stimulus interval of 6 s, 8 s or 10 s. Additionally, 21 silent trials (24 in the second and third fMRI experiment) were presented pseudo-randomly throughout the experiment with a duration of 20 s each.

The participants had to categorize the FM tones according to the direction of modulation. They had to press a button with the right index finger in response to upward modulated FM tones and another button with their right middle finger indicating downward modulated FM tones.

During the entire experiment participants had to look at a white fixation cross on a grey background. During the fMRI experiments this visual presentation was projected onto a screen which could be viewed via a mirror mounted on the head coil. In the first fMRI experiment, the feedback indicated the correctness of the participants’ response. A green checkmark was presented for 500 ms, if the participants answered correctly and within 1.5 s after FM tone onset. If they pressed the wrong button or answered too slowly, a red cross was presented for 500 ms. In the second and third fMRI experiment, subjects received registering feedback (green checkmark) that only indicated that the system has registered their response without evaluating the correctness of their input. Again, if they answered too slowly, a red cross was presented. Trials, in which participants missed to press the button in the appropriate time, were excluded from further analysis.

Delay times: In the first fMRI experiment, participants received immediate feedback subsequent to their button press in 85% of all trials. In 15% this feedback was delayed by 200 ms (5%), 400 ms (5%), or 600 ms (5%). In the second and third fMRI experiment, delays in feedback presentation were presented pseudo-randomly and equally often as immediate feedback with an average delay duration of 500 ms (300 ms, 500 ms, 700 ms). Additionally, during the third experiment feedback was omitted in 10% of all trials.

### Posttest

To control for individual differences in perceiving a delay, we determined the just noticeable delay of each participant of our first fMRI experiment in a subsequent session. In a quiet room at a PC participants had to categorize the same upward and downward modulated FM tones and pay attention to the timing of the visual feedback. This posttest was subdivided in blocks of eight FM stimuli. The inter trial interval was 1 s. Never, or only once in a block the feedback was delayed. Between each block subjects had to indicate by keystroke if they perceived a delay (“v”) or not (“n”). Starting with a delay of 800 ms, the duration of the delay was adaptively adjusted according to the participants’ answer in steps of 50 ms. If they detect a delay correctly the duration of the subsequent delay was shortened by 50 ms. If they did not detect the presented delay the subsequent delay was prolonged by 50 ms. In case the duration of the delay fell below 300 ms or if participants completed 20 blocks we narrowed the step range to 25 ms. At that time subjects had to solve another 30 blocks in which the just noticeable delays leveled off. The measurement took about 30 min.

### Data acquisition -fMRI

The measurements of all three fMRI experiments were carried out in a 3 Tesla scanner (Siemens Trio, Erlangen, Germany) equipped with an eight channel head coil. A 3D anatomical data set of the participant’s brain (echo time (TE), 4.77 ms; repetition time (TR), 2500 ms; flip angle,7° matrix size, 256×256; field of view, 25.6×25.6 cm; 192 slices of 1 mm each) was obtained before the functional measurement. Additionally, an Inversion-Recovery-Echo-Planar-Imaging (IR-EPI) was acquired that has the same geometric distortions as the functional measurement but a reversed contrast and thus serves the purpose of a more precise co-registration of the functional data to the anatomical data. For the first fMRI experiment, 1290 functional volumes were acquired in 43 min using an echo planar imaging (EPI) sequence (TE, 30 ms; TR, 2000 ms; flip angle, 80°; 3 mm isotropic resolution, 32 slices, 0.3 mm gaps). In the second and third fMRI experiments 843 functional volumes were acquired in 28 min and 6 s.

The head of the participant was fixed with a cushion with attached ear muffs containing the fMRI compatible headphones [11]. Additionally, the participants wore earplugs. The software Presentation (Neurobehavioral Systems, Albany, USA) was used for stimulus presentation and recording behavioral responses. Before the experiment, the overall stimulus intensity was adjusted for each participant to a comfortable level and equally loud at both ears. Visual stimuli were presented by a video projector onto a back projection screen, which was visible inside the scanner via a mirror system.

### Data analysis -fMRI

The functional data were analyzed with the software BrainVoyager™ QX (Brain Innovation, Maastricht, The Netherlands). A standard sequence of preprocessing steps, i.e., slice scan time correction, 3D-motion correction, linear trend removal, spatial smoothing with a Gaussian filter of 4 mm full width at half maximum, and temporal filtering with a high-pass of three cycles per scan was performed. The functional data were co-registered with the 3D anatomical data by utilizing the IR-EPI, and then transformed into Talairach-space.

The functional data were z-transformed. To obtain a better estimate of the hemodynamic response function we employed the deconvolution analysis implemented in BrainVoyager. Here the hemodynamic response is not estimated from a fixed function (like a γ-function) but is flexibly and adaptively estimated from the data. The deconvolution analysis models the hemodynamic response function based on the points in time t when a stimulus is presented, under the assumption of linearity and a finite number of data points of the response as predictors. For each condition ten points in time (18 s, predictors) were defined. The conditions were immediate feedback, 200 ms delay, 400 ms delay, and 600 ms delay and each of these were divided into correct and false responses. In the first fMRI experiment the resulting design matrix X consisted of 81 columns, ten columns (predictors) for each condition and the constant factor k (mean gray value) that represented the baseline. Accordingly, 81 beta weights were estimated that allowed the reconstruction of the hemodynamic BOLD response for each condition.

To identify the regions with differential BOLD responses, we compared the conditions at the conjoined time points 4 and 5 in a multi-subject GLM using a random effects analysis. Thus, the analysis focused on the period from 6 to 10 s after stimulus onset. The reported general linear model (GLM) parameters (beta weights) provide a direct estimate of the actual percent signal change. To identify regions that were differentially activated by delayed vs. immediate feedback, we computed three contrasts that compared each delay condition (200 ms, 400 ms, and 600 ms) against the condition with immediate feedback (q(FDR) = 0.05: t_200ms_ = 4.20, t_400ms_ = 5.00, t_600ms_ = 4.42). Only trials with correct responses (positive feedback) were considered because false responses were rare and did not occur in all subjects and conditions. For each contrast volumes-of-interest (VOIs) were defined for all resulting clusters that comprised at least 27 mm^3^. The size of these VOIs was determined by counting the number of enclosed voxels. To reduce signal artefacts from brain areas with low signal intensity, only those voxels were considered whose functional EPI signal had a grey level of at least 75. For each VOI, a random effect ROI-GLM (region of interest-general linear model) as implemented in BrainVoyager was conducted to determine the mean beta-value of each condition.

Mean reaction times and error rates of each participant were calculated from the behavioral log data of the fMRI experiments. These behavioral data was imported to SPSS (IBM Corporation, New York, USA) and examined for normal distribution using the Kolmogorov-Smirnov test.

In the second and third fMRI experiment, we first computed the contrast between delayed and immediate feedback (RFX-analysis). Trials in which participants answered too slowly or missed to answer were excluded from this analysis. In a further step, the main ROIs of the first fMRI experiment (bilateral anterior insula, posterior medial frontal cortex, left inferior parietal lobule, right inferior frontal junction) were used to calculate the BOLD response for each of those regions in the second and third fMRI experiment.

## Results

### Experiment 1

The first fMRI experiment aimed to clarify if delays at or below a just noticeable delay already affect brain activity and if the impact of a delay on brain activity increases with the duration of the delay. According to a previous study [8] we expected to find stronger activity in a network of brain regions that comprise the posterior medial frontal cortex, anterior insula, inferior frontal gyrus, and inferior parietal lobule in response to delayed feedback compared to immediate feedback. However, this previous study used delays of 500 ms. Guidelines in human-computer interaction research assume that systems in simple repetitive interaction tasks should behave like physical devices and respond at least in 200 ms [1,4]. Therefore, we wanted to investigate if unexpected delays of 200 ms are already sufficient to increase the activity in a network of brain regions. Besides these 5% of trials with unexpected delays of 200 ms, we delayed another 5% of trials by 400 ms and 5% of trials by 600 ms. We hypothesized that the activity in the network of brain regions increases with the duration of the delay. However, we had to verify if subjects already perceive such short delays of 200 to 600 ms while solving an auditory categorization task. Therefore, they participated in a posttest where we adaptively tested their just noticeable delay under the same task conditions as in the fMRI measurement.

### Results–behavioral data

The mean just noticeable delay of the participants of our first fMRI experiment was 327.2 ms ± 89.7. This result allows the conclusion that delays of 200 ms as used in the context of our fMRI experiment are well below the threshold of perceiving a delay. Delays of 400 ms lie in the range of a just noticeable delay and delays of 600 ms lie above this threshold. The mean error rate of the auditory categorization task in the posttest was 18.0% ± 7.0.

The mean error rate during the fMRI experiment was 16.1% ± 8.3.

The evaluation of the questionnaire revealed that five subjects did not perceive any delay during the fMRI experiment. These five subjects neither differ in their behavioral data from the rest of the group nor did we find an increased threshold of a just noticeable delay.

### Results–fMRI data

The contrast between trials with feedback delays of 600 ms vs. immediate feedback revealed a network of brain regions (see Table 1). We found stronger activation by delayed feedback most prominent in the left and right anterior insular cortex (aI), in the posterior medial frontal cortex (pMFC), the left inferior parietal lobule (LPI), and in the right inferior frontal junction (IFJ). Furthermore, we observed a significant decrease in the BOLD response in parts of the default network [12] (medial prefrontal and posterior cingulate cortex) which occur when the participants’ attention is drawn from internal processes to external tasks [12–14] (see Table 1).

**Table 1:** First fMRI-Experiment: Regions with stronger activity during delayed (600 ms) compared to immediate feedback (FDR < 0.05).

Region of activation	hemisphere	BA	Talairach			Volume	Mean t
			x	y	z		
**anterior insular cortex**	right	13	34	19	6	1914	5.33
**anterior insular cortex**	left	13	-30	20	6	511	4.86
**posterior medial frontal cortex**	right	6/32	5	9	47	439	4.87
**inferior parietal lobule**	left	40	-49	-32	44	279	4.89
**inferior frontal junction**	right	9	45	7	31	613	4.74
**Insula**	left	13	-37	-4	18	99	4.87
**Thalamus**	right		9	-11	10	983	5.26
**Thalamus**	left		-9	-15	9	1005	5.41
**fusiform gyrus**	right	37	29	-43	-14	110	4.85
**Region of deactivation**							
**posterior cingulate cortex**	left	7	-2	-57	32	288	-4.85
**medial frontal gyrus**	left	10	-6	56	16	235	-4.87
**middle temporal gyrus**	left	39	-43	-62	27	85	-4.60

**Fig 1:**
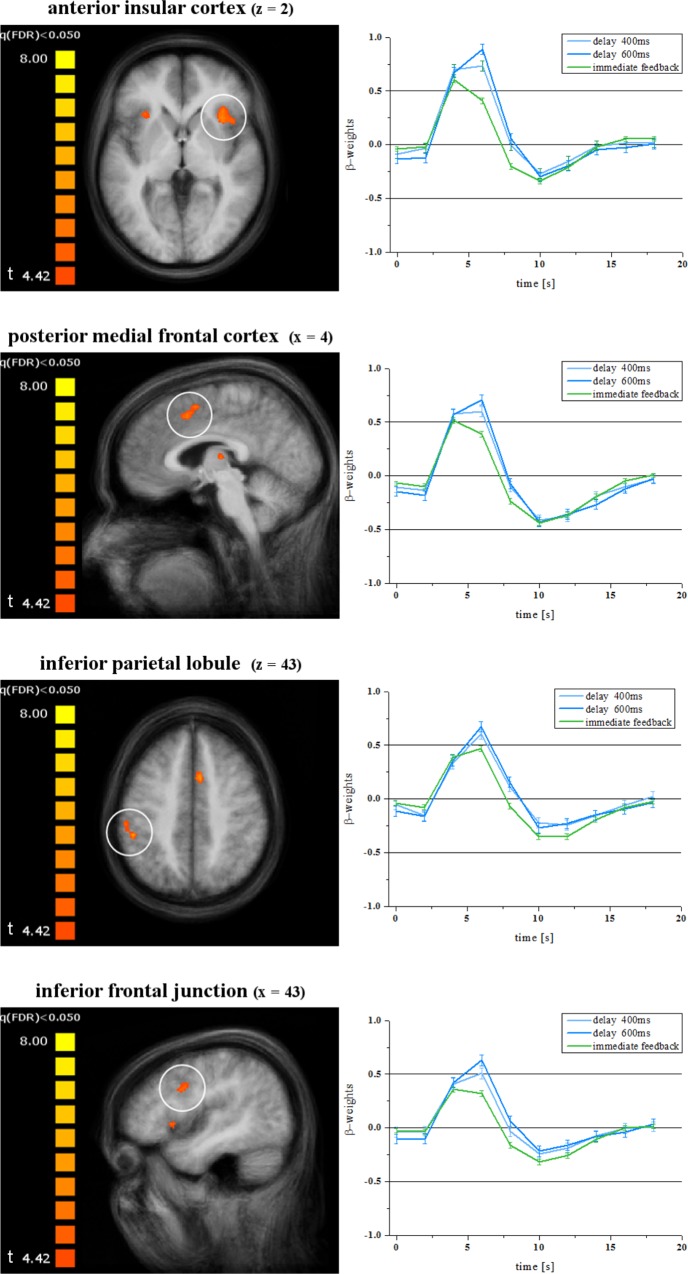
Experiment 1 –Activity in the anterior insular cortex, posterior medial frontal cortex, left inferior parietal lobule and right inferior frontal junction. Regions with stronger activation in response to delays of 600 ms or 400 ms each compared to the immediate feedback (left side: activation clusters, right side: BOLD time course). Delays of 200 ms are not significantly different compared to immediate feedback. Error bars indicate standard errors of the mean.

The contrast between delays of 400 ms and immediate feedback resulted in fewer regions of significant activation differences (see Table 2). Overlapping with the regions activated by 600 ms delay vs. immediate feedback were bilateral anterior insular cortex, the posterior medial frontal cortex, the left inferior parietal lobule and the right inferior frontal junction. For these regions Fig 1 shows the location as well as the respective BOLD response for the feedback conditions. Note that the contrast between delays of 200 ms and immediate feedback showed no significant differences.

**Table 2:** First fMRI-Experiment: Regions with stronger activity during delayed (400 ms) compared to immediate feedback (FDR < 0.05).

Region of activation	hemisphere	BA	Talairach			Volume	Mean t
			x	y	z		
**anterior insular cortex**	right	13	34	22	6	827	5.77
**anterior insular cortex**	left	13	-33	18	7	738	5.82
**posterior medial frontal cortex**	right	6/32	6	20	38	45	5.64
**inferior parietal lobule**	left	40	-36	-39	49	48	5.37
**inferior frontal junction**	right	9	45	3	30	106	5.24
**postcentral gyrus**	left	2	-52	-23	38	80	5.32

**Fig 2:**
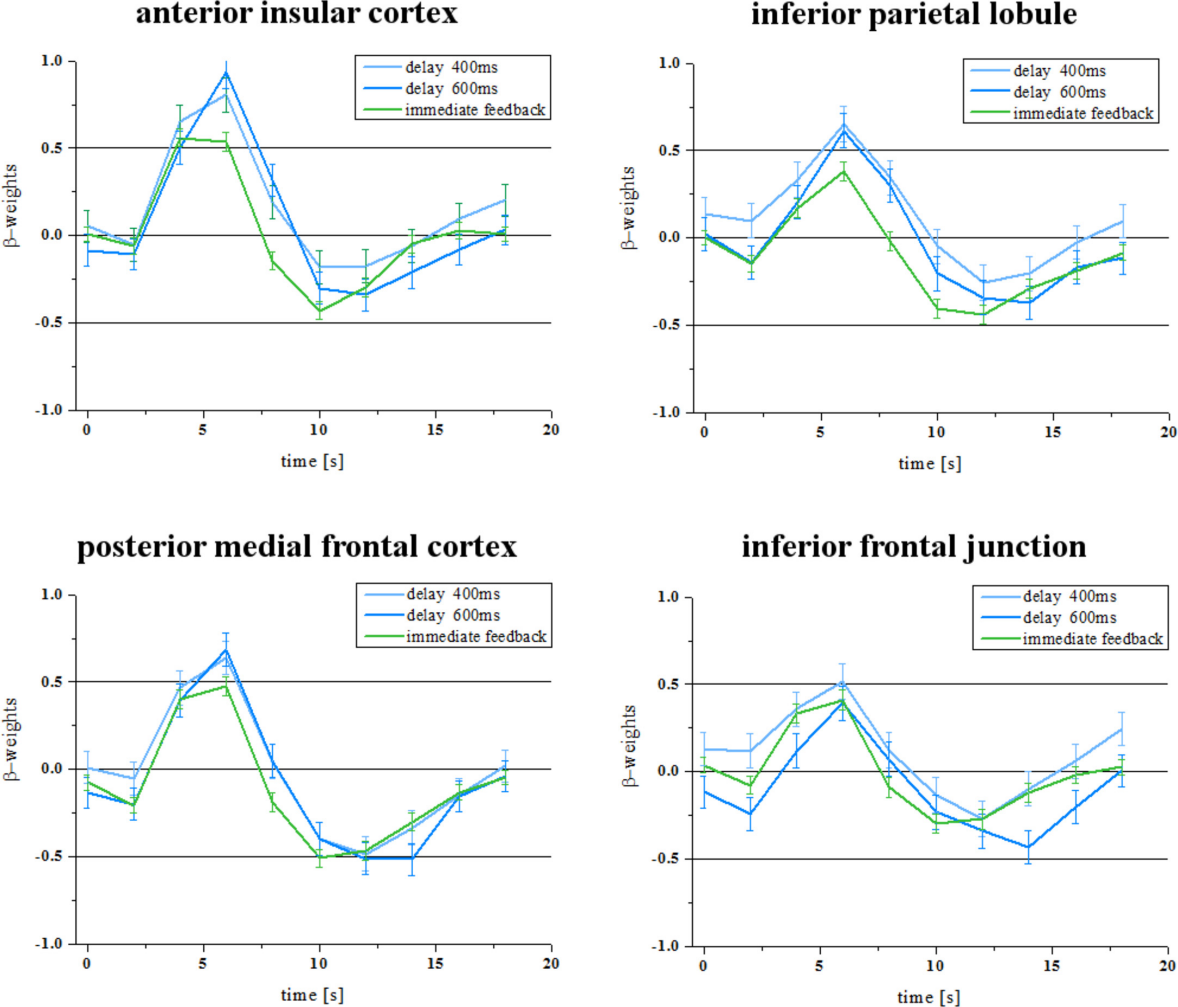
Experiment 1 –five participants. BOLD time courses from the ROIs shown in Fig 1 for those participants who did not report to have noticed the delays in feedback after the experiment.

In addition, we separately analyzed the five subjects who did not report to have noticed the delays in feedback after the experiment. However, we found largely comparable results as compared to the group analysis with respect to the activated brain areas and the BOLD response of the ROI as defined in experiment 1 (except for the inferior frontal junction) (Fig 2).

### Experiment 2 and 3

In the first fMRI experiment we only used unexpected–infrequently occurring delays. Shneiderman and Plaisant [4], however, proposed that users of technical systems are able to adapt their behavior to regular (constant) delays. Therefore, we examined if these changes on a behavioral level are accompanied by changes in brain activity. We hypothesized that the observed increase in brain activity in response to unexpected delays decreases when delays occur frequently.

### Results–behavioral data

The evaluation of the questionnaires revealed that 40% of the participants of the second experiment and 30% of the participants of the third experiment had not perceived any delay in feedback presentation or at least could not remember any delays. Furthermore, 53% of the participants of the third experiment reported that they did not realize that the omission of feedback is part of the experiment.

### Results–fMRT data–Experiment 2

**Fig 3:**
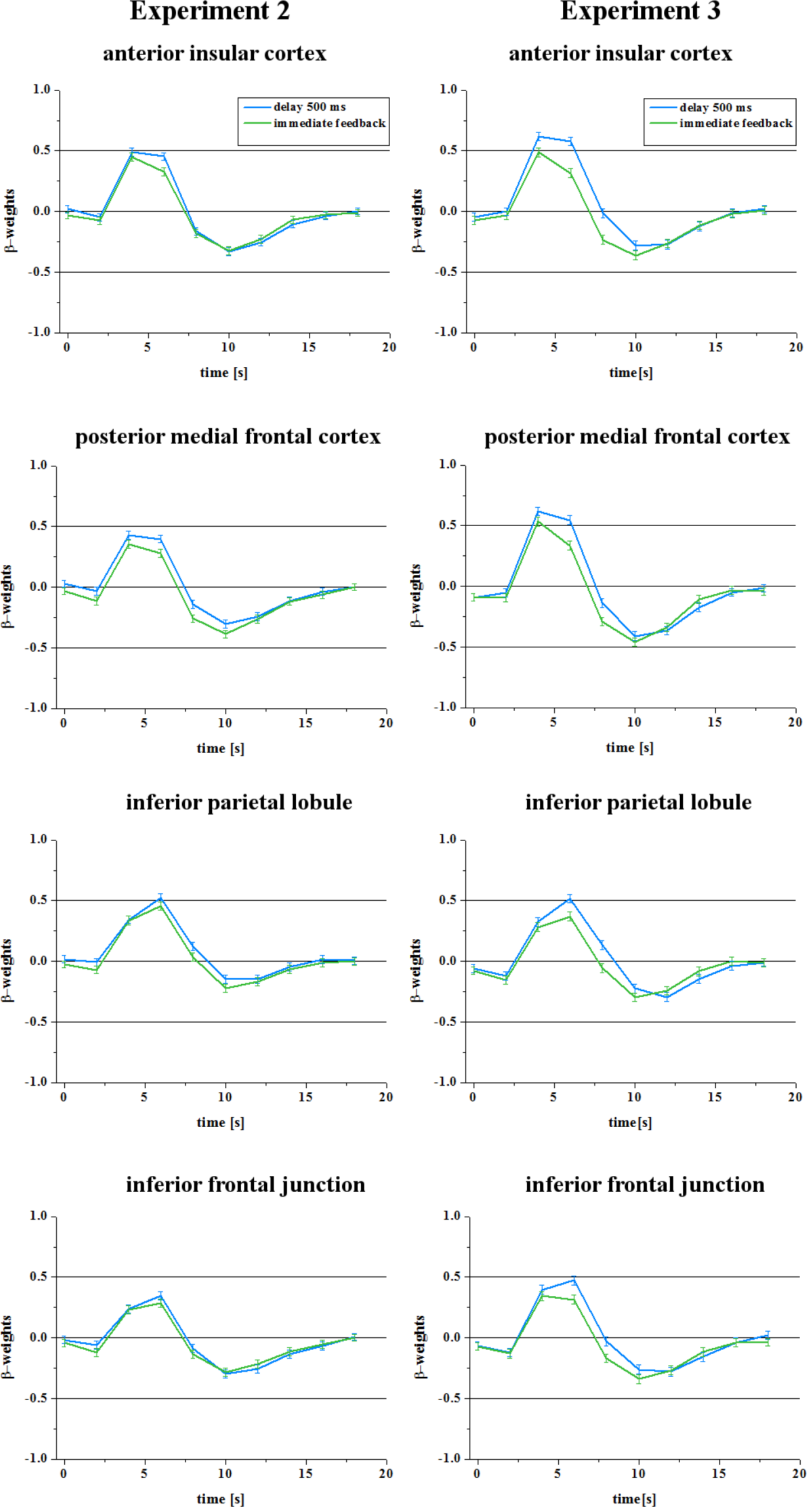
BOLD response of ROIs in the second and third experiment. In the second experiment (left column) delayed feedback did not lead to a significant increase of the BOLD response in four regions of interest as defined in experiment 1. However, in the third experiment, which included a few omissions of feedback, the delays in feedback led to a significant increase in BOLD response in these regions (right column).

The analysis of the direct contrast between delayed feedback and immediate feedback revealed no significant differences at the chosen significance level (q(FDR) = 0.05). In Fig 3 (left column) we show the time course of the BOLD response of the ROIs as defined in experiment 1 which confirms the similarity in activation elicited by immediate and delayed feedback compared to the effect shown in Fig 1. Therefore, we can assume that users of technical systems can adapt to frequent delays.

### Results–fMRT data–Experiment 3

The direct contrast between delayed feedback compared to immediate feedback led to a significant increase in BOLD response within the bilateral anterior insula, posterior medial frontal cortex, and left inferior parietal lobule. Due to the minimum cluster threshold of 27 mm^3^ we did not find activity differences within the right inferior frontal junction. However, we still found an increase in activity in response to delayed feedback in the right inferior fontal gyrus and further regions (see Table 3).

**Table 3:** Third fMRI-Experiment: Regions with stronger activity during delayed compared to immediate feedback (FDR < 0.05).

Region of activation	hemisphere	BA	Talairach			Volume	Mean t
			x	y	z		
**anterior insular cortex**	right	13	34	18	4	316	5.08
**anterior insular cortex**	left	13	-34	16	5	118	5.05
**posterior medial frontal cortex**	right	6/32	3	8	46	320	5.20
**inferior/superior parietal lobule**	left	7	-33	-50	48	386	5.45
**superior parietal lobule**	right	7	27	-59	43	102	5.22
**inferior frontal gyrus**	right	44	52	6	15	86	5.09
**precuneus**	right	7	13	-61	37	362	5.36
**precuneus**	left	7	-22	-60	49	118	5.27

In Fig 3 (right column) we show the time course of the BOLD response of the ROIs as defined in experiment 1 which confirms that even frequent delays in feedback presentation significantly increase the activity in those regions when they occur together with omissions of feedback.

## Discussion

The series of fMRI experiments revealed that neural activation of brain regions, known to be involved in attentional and action control [13,14], nicely match with behavioral indicators of potentially adverse effects of delays in human-computer interaction [2]. Whereas delays that on average are below a just noticeable threshold (200 ms) do not lead to a significant increase in activation, those above such a threshold (400 ms, 600 ms) do lead to a strong recruitment of the bilateral anterior insular cortex, posterior medial frontal cortex, inferior parietal lobule and inferior frontal junction. Thus, the observed increase of activity in these brain regions can be taken as indicator of an unsuccessful human-computer interaction at the moment of the occurrence of unexpected delays. The results of our second fMRI experiment demonstrate that frequently occurring delays initiate processes of adaptation. During the course of the interaction the user’s temporal expectation about the action outcome is adjusted, the additional neural resources in attention and action control are no longer required and the differences in brain activity between frequently delayed and immediate feedback are no longer detectable. This is in line with the assumption of Shneiderman and Plaisant [4] that users of technical systems are able to adapt to regularly occurring delays for instance by changing their working style. For that reason, software ergonomics sometimes recommend to present system responses with regular and constant delays with the intention to change the users’ temporal expectation of feedback presentation [3,4]. However, our results show that it is highly important to make sure that the system will not fail completely during the course of the interaction. Otherwise any delay in feedback that occurs after a failure trial may be treated as an indicator of another potential breakdown of the interaction because during the delay duration the user cannot be certain whether or not the feedback will occur. This prevents the neural adaptation process that we observed in sessions with regular delays but without failures.

Beyond this general interpretation of the overall results of the three fMRI experiments, which insights do the results provide regarding the function of the identified network?

Several studies indicate that the pMFC is activated by internal and external error signals [15–18] especially after the nonoccurrence of a predicted outcome [19,20]. However, recent studies show that this brain region is also activated in response to any conflictual event [17,20–22]. This more general function is supported by our findings. It also fits to the idea that any deviation from a temporal expectation of an action outcome leads to a shift in attention and triggers an orienting response evident, i.e., in an increased P300 response [7], which is a typical component of an organisms’ orienting response [13,23,24]. Furthermore, early psychophysiological studies [6,25] and a recent study of Kohrs et al. [5] could show that delays in system response are accompanied by an increase in skin conductance, a further typical component of an orienting response. The strength of such an orienting response depends on the relevance for the organism [26]. Several studies show that this autonomic response is correlated to the pMFC and anterior insula activity [20,27–29]. According to Cieslik et al. [30] the anterior insula and the pMFC play a central role in supervisory attentional control. Especially the pMFC seems to monitor task performance and is associated with the “energization of the relevant, non-dominant task schema” by switching from automatic to controlled responses [30]. Presumably, delays initiate processes of action planning and might shift the users’ attention to the repetition of their initial action. The stronger activation of the left inferior parietal lobule may reflect such processes too as it is known to be activated whenever motor attention is needed [31,32]. Furthermore, the inferior parietal lobule is activated during attentional reorientation and redirections of response intentions [30,31] and sends integrated sensory input and motor plans to the pre-supplementary motor area/pMFC [33,34].

It is under debate at which stage consciousness comes into play. Whereas some studies suggest that activity in the anterior insula (and pMFC) is stronger when subjects consciously perceive a salient event such as an error [35], others did not find a difference between consciously and unconsciously perceived errors [36] (for an overview see Ullsperger et al. [20]). Most theories on predictive coding do not imply a conscious engagement of the subject in the comparison of expectations and new input [33]. According to Jakobs et al. [33] the validity of the environment primarily influences the neural activity while the subjective evaluation of the situation has less influence. This view fits well with our finding of a comparable increase in activation by delayed feedback in the anterior insula and pMFC of those subjects who could not remember any occurrence of feedback delays. Even more so, the strong activation of brain areas involved in attentional and action control emphasizes the relevance of a smooth communication in human computer interaction because any unexpected delay above a certain threshold elicits brain activity that may distract the user from reaching the current goal.
